# Crowned dens syndrome

**DOI:** 10.1002/ccr3.3058

**Published:** 2020-06-23

**Authors:** Vincent Zimmer

**Affiliations:** ^1^ Department of Medicine Marienhausklinik St. Josef Kohlhof Neunkirchen Germany; ^2^ Department of Medicine II Saarland University Medical Center Saarland University Homburg Germany

**Keywords:** calcium pyrophosphate deposition, disease, fever of unknown origin, meningoencephalitis, pseudogout, systemic inflammation

## Abstract

Crowned dens syndrome (CDS) is rare, though likely underdiagnosed, typically presenting with fever, neck pain, and stiffness. Adequate clinical awareness may streamline diagnostics and therapeutics and avoid the need for invasive procedures.

## Graphical abstract

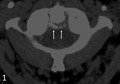

A 95‐year‐old male with stable vital signs presented to the emergency department for neck pain and febrile temperatures of 38.7°C. Clinical examination was significant for neck stiffness and pain with restricted rotational movement. There were no signs for neurologic deficits and/or alternative potentially infectious foci. The systemic inflammatory marker C reactive protein was elevated to 18.3 mg/dL with a normal procalcitonin. We prioritized computed tomography (CT) of the cervical spine as a noninvasive modality over lumbar puncture. CT indicated curvilinear periodontoid calcifications of the transverse ligament of the atlas on axial (Figure 1) and sagittal (Figure 2) planes. In an appropriate clinical setting, this warrants a clinical diagnosis of crowned dens syndrome (CDS) as crown‐ or halo‐shaped calcium pyrophosphate dihyrate (CPPD) or hydroxyapatite depositions surrounding the odontoid process. CDS is considered an underdiagnosed, yet typical presentation of calcium pyrophosphate deposition disease (CPDD) as the second most common crystal‐induced arthritis.^1^ Therapeutic strategies lean on gout management, including nonsteroidal anti‐inflammatory drugs (NSAIDs), colchicine, or low‐dose steroids in chronic kidney disease.

**FIGURE 1:**
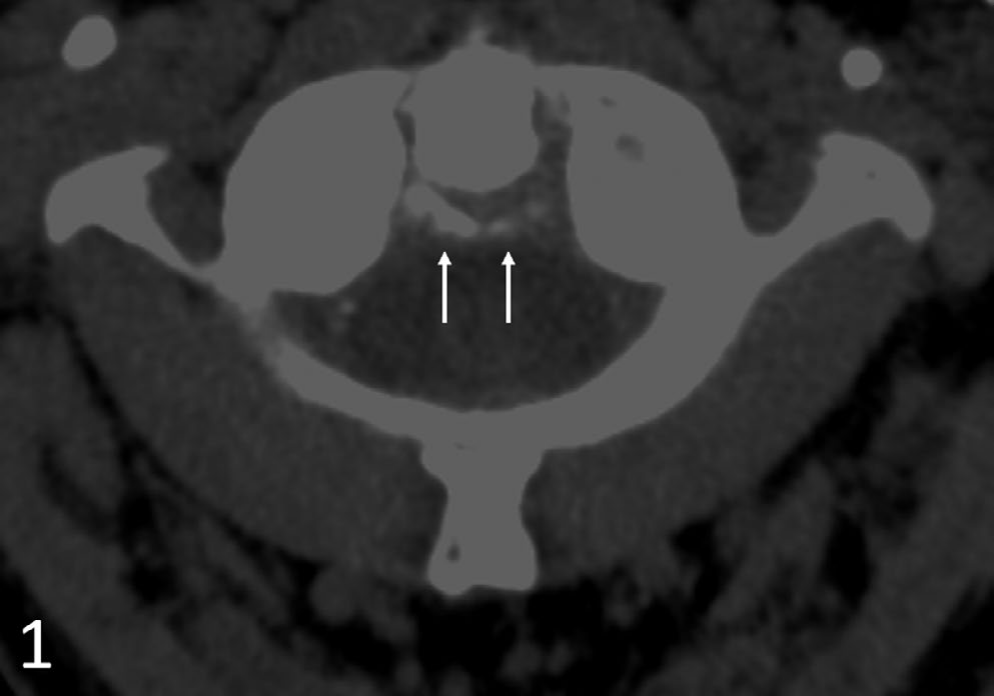
Axial CT of the cervical spine indicating linear calcifications of the transverse ligament of the atlas

**FIGURE 2:**
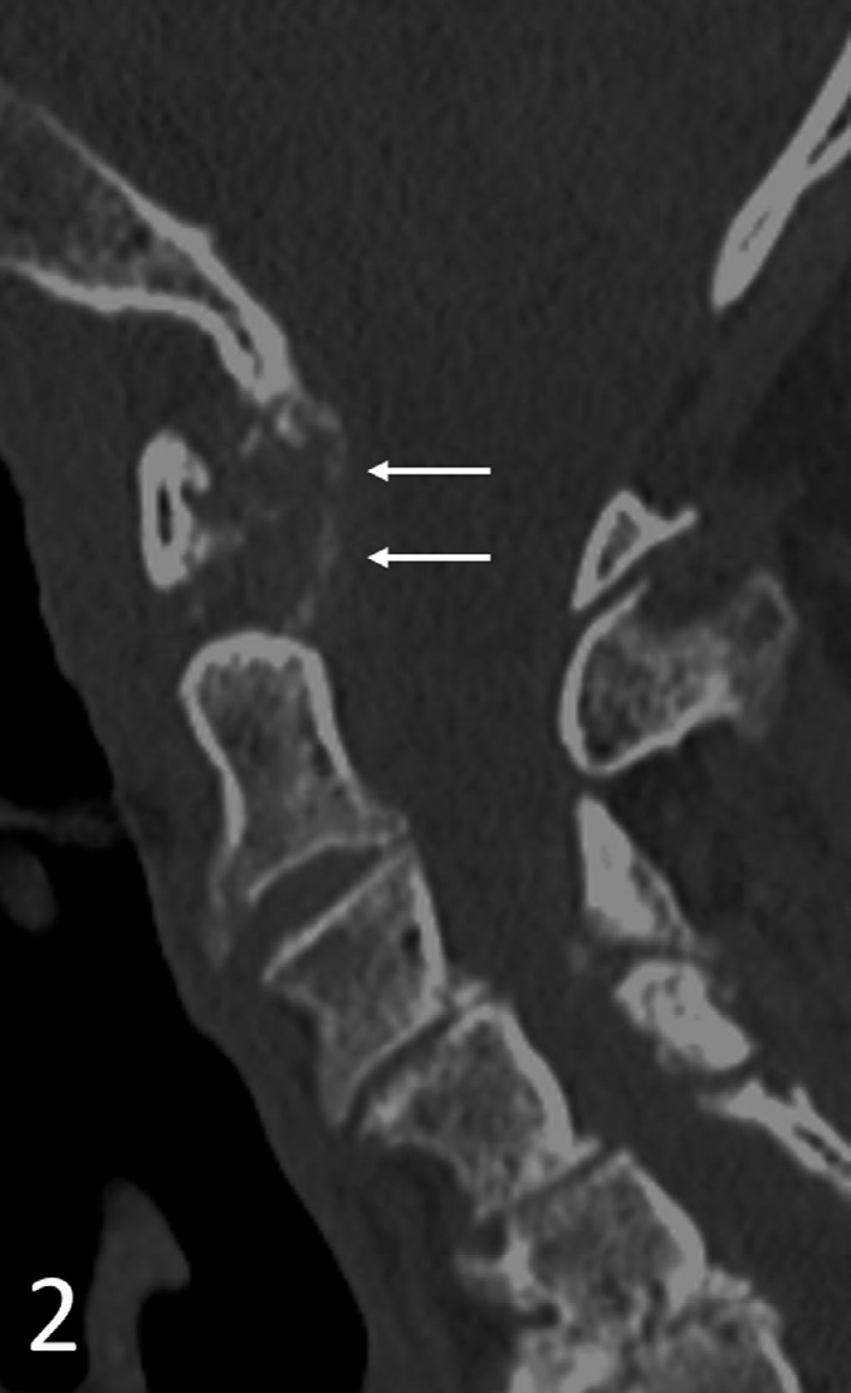
Sagittal images likewise indicate curvilinear periodontoid calcifications in the transverse ligament and the atlantoaxial space

Acute presentations of CDS are common and challenging without an established diagnosis of CPDD/chondrocalcinosis outside the atlantoaxial junction and may be presenting as acute neurologic emergencies, such as bacterial meningitis, or rheumatological conditions, such as polymyalgia rheumatica.^2^

## CONFLICT OF INTEREST

None declared.

## AUTHOR CONTRIBUTIONS

VZ: Clinical care, drafting and finalization of manuscript.

## References

[1] Haikal A , Everist BM , Jetanalin P , Maz M . Cervical CT‐dependent diagnosis of crowned dens syndrome in calcium pyrophosphate dihydrate crystal deposition disease. Am J Med. 2020;133(2):e32‐e37. 31369722 10.1016/j.amjmed.2019.06.050

[2] Shikino K , Ota T , Ikusaka M . Crowned dens syndrome. Am J Med. 2017;130(3):e111‐e112. 28215951 10.1016/j.amjmed.2016.10.026

